# Painful and non-painful chemotherapy-induced peripheral neuropathy and quality of life in colorectal cancer survivors: results from the population-based PROFILES registry

**DOI:** 10.1007/s00520-020-05438-5

**Published:** 2020-04-12

**Authors:** C. S. Bonhof, H. R. Trompetter, G. Vreugdenhil, L. V. van de Poll-Franse, F. Mols

**Affiliations:** 1 CoRPS - Center of Research on Psychology in Somatic Disorders, Department of Medical and Clinical Psychology, Tilburg University, PO Box 90153, 5000 LE Tilburg, the Netherlands; 2 Department of Research, Netherlands Comprehensive Cancer Organisation (IKNL), Utrecht, The Netherlands; 3 Department of Internal Medicine, Máxima Medical Centre, Eindhoven and Veldhoven, The Netherlands; 4 Division of Psychosocial Research and Epidemiology, The Netherlands Cancer Institute, Amsterdam, The Netherlands

**Keywords:** Chemotherapy-induced peripheral neuropathy, Colorectal cancer, Health-related quality of life, Numbness, Pain, Tingling

## Abstract

**Purpose:**

This study aims to (1) examine the prevalence of painful versus non-painful chemotherapy-induced peripheral neuropathy (CIPN) among long-term colorectal cancer (CRC) survivors, (2) identify sociodemographic, clinical, and psychological factors associated with painful and non-painful CIPN, and (3) examine the associations of painful CIPN with health-related quality of life (HRQoL) in comparison with non-painful CIPN, i.e., numbness/tingling.

**Methods:**

All CRC survivors diagnosed between 2000 and 2009 as registered by the population-based Netherlands Cancer Registry (Eindhoven region) were eligible for participation. Chemotherapy-treated survivors (*n* = 477) completed questions on CIPN (EORTC QLQ-CIPN20) and HRQoL (EORTC QLQ-C30).

**Results:**

Painful CIPN was reported by 9% (*n* = 45) of survivors and non-painful CIPN was reported by 22% (*n* = 103). Time since diagnosis was related to painful CIPN, and time since diagnosis, a higher disease stage, osteoarthritis, and more anxiety symptoms were related to non-painful CIPN. Finally, survivors with painful CIPN reported a worse global quality of life and worse physical, role, cognitive, and social functioning compared to survivors with non-painful CIPN and those without any sensory CIPN. No differences were found between survivors with non-painful CIPN and those without sensory CIPN.

**Conclusions:**

It seems that painful CIPN must be distinguished from non-painful CIPN, as only painful CIPN was related to a worse HRQoL. Future research is needed to examine whether painful CIPN must be distinguished from non-painful CIPN regarding predictors, mechanisms, and treatment.

## Introduction

Chemotherapy has contributed significantly to the increased survival rates in colorectal cancer (CRC) [1]. However, patients frequently live with the long-term side effects of this treatment. Chemotherapeutic agents commonly used in the treatment of CRC are highly toxic to the peripheral nervous system. As a result, patients often develop chemotherapy-induced peripheral neuropathy (CIPN) [2]. CIPN most commonly presents as sensory neuropathy, with symptoms such as tingling, numbness, and burning pain in the hands or feet in a characteristic stocking-glove distribution [2]. Unfortunately, there are currently no agents available for the prevention or treatment of CIPN, and so, dose reduction and even cessation of treatment are often necessary to prevent severe CIPN. After chemotherapy, CIPN symptoms often improve or even disappear, but it is a chronic issue for about 30% of patients [3].

A recent review showed that CIPN severely impacts patients’ health-related quality of life (HRQoL) [4]. In a previous PROFILES study using the same sample as the current study, it was found that only sensory neuropathy symptoms significantly distinguished chemotherapy-treated survivors from those who did not receive chemotherapy and that these symptoms were associated with worse scores on all the EORTC QLQ-C30 scales [5]. However, sensory neuropathy consists of a broad range of symptoms, and little is known about the specific sensory neuropathy symptoms and their independent impact on HRQoL. In the previously mentioned PROFILES study, it was shown that only the sensory neuropathy symptoms tingling, numbness, and shooting or burning pain were more often reported by survivors with chemotherapy compared to survivors who did not receive chemotherapy [5]. While numbness and tingling are found to be highly correlated [6, 7], correlations between painful CIPN and either numbness or tingling appear to be much weaker [7]. Also, severe numbness and tingling commonly exist without neuropathic pain symptoms, while the reverse is not common [7, 8]. These findings indicate that painful CIPN must be evaluated separately from non-painful CIPN symptoms, such as numbness and tingling.

In diabetes research, studies on diabetic peripheral neuropathy (DPN) already make an explicit distinction between painful and non-painful DPN. Pain is the main symptom causing patients with DPN to seek help [9] and results in more severe impairments in HRQoL compared to non-painful DPN [10, 11]. In cancer research, few studies have focused on the difference between painful and non-painful CIPN. A study among patients treated with adjuvant oxaliplatin or docetaxel found that among patients treated with docetaxel, those with persistent pain had significantly less improvement in anxiety and depression over time than those without persistent pain [12]. Another study among patients with colorectal, breast, lung, or prostate cancer showed that baseline neuropathic pain, but not numbness and tingling, was associated with more than twice the odds of significantly declining HRQoL in CRC patients, but not in those with other cancer types [13]. However, these studies have only examined the effect of painful versus non-painful CIPN on patient-reported outcomes up to 1 year after diagnosis, while it is unknown whether the effect is still present among long-term survivors.

Extending our knowledge on the difference between painful and non-painful CIPN seems warranted, as it could help future trials on the treatment of CIPN to determine its specific target. In addition, it is needed to identify those at high risk of developing a low HRQoL. Furthermore, gaining more insight into the correlates of (non-)painful CIPN is important to develop personalized management of CIPN. Therefore, the aims of this study were to (1) examine the prevalence of painful versus non-painful (i.e., numbness and tingling) peripheral neuropathy symptoms in chemotherapy-treated CRC survivors 2–12 years after diagnosis, (2) identify sociodemographic, clinical, and psychological factors (i.e., anxiety and depressive symptoms) associated with the presence of painful and non-painful CIPN, and (3) examine the associations of painful CIPN with HRQoL in comparison with non-painful CIPN.

## Methods

### Settings and participants

Details of the data collection process have previously been described [5]. In the current study, additional details of clinical characteristics were available, due to the regular updates of our cancer registry. The survey was set up in December 2010, and patients who were eligible for participation were selected from the Netherlands Cancer Registry, which routinely collects data from all individuals newly diagnosed with cancer in the Netherlands [14]. For this study, all individuals from the Eindhoven region (> 2 million inhabitants) who were diagnosed with CRC between 2000 and 2009 were eligible for participation. Survivors who had cognitive impairment, who died prior to the start of the study or were terminally ill, those with carcinoma in situ, those already included in another study, and those with unverifiable addresses were excluded. One year later, the second data collection wave took place. For this study, data from this second wave was used, as it included a questionnaire on CIPN. At this time, patients were diagnosed 2–12 years ago. This study was approved by a certified medical ethics committee. All patients signed informed consent.

### Data collection

Data collection was performed within PROFILES (Patient Reported Outcomes Following Initial Treatment and Long Term Evaluation of Survivorship), a registry for the physical and psychosocial impact of cancer and its treatment [15]. Eligible CRC survivors were informed of the study via a letter from their (ex-) attending specialist. A reminder letter was sent to non-respondents within 2 months.

### Study measures

#### Sociodemographic and clinical characteristics

Survivor’s sociodemographic (i.e., age, sex) and clinical information (e.g., date of diagnosis, tumor stage, and treatment) was obtained from the NCR. The adapted Self-administered Comorbidity Questionnaire (SCQ) [16] was used to assess comorbidity at time of the study. Questions on partner status and educational level were added to the questionnaire.

#### Psychological factors

Symptoms of anxiety and depression were assessed with the Hospital Anxiety and Depression Scale (HADS) [17]. It consists of 14 items: 7 items measure depressive symptoms and the other 7 items measure anxiety. Items are answered on a four-point Likert scale and the total score for each scale ranges from 0 to 21, with higher scores indicating more psychological distress.

#### Chemotherapy-induced peripheral neuropathy

The sensory scale of the European Organization for Research and Treatment of Cancer Quality of life Questionnaire Chemotherapy-Induced Peripheral Neuropathy 20 (EORTC QLQ-CIPN20) [18] was used to assess chemotherapy-induced peripheral neuropathy. Respondents are asked to indicate how often they had experienced the specific symptom in the past week. Items are answered on a four-point Likert scale ranging from (1) *not at all* to (4) *very much*. In the previous PROFILES study using the same sample as the current study [5], we found that survivors treated with chemotherapy only reported more shooting/burning pain, numbness, and tingling compared to survivors not treated with chemotherapy. On all other EORTC QLQ-CIPN20 items, there were no differences between the two groups. Therefore, we will only focus on these three symptoms, as we are specifically interested in peripheral neuropathy caused by chemotherapy. Painful CIPN was assessed using the items on shooting or burning pain in the (1) fingers or hands and (2) toes or feet. If survivors answered at least one of the items with “quite a bit” or “very much,” painful CIPN was considered to be present. The items on (1) tingling fingers or hands, (2) tingling toes or feet, (3) numbness in fingers or hands, and (4) numbness in toes or feet were grouped together as an assessment of non-painful CIPN and considered to be present if survivors answered at least one of these items with “quite a bit” or “very much.” Finally, survivors who did not report painful or non-painful CIPN were considered to have “no sensory CIPN.”

#### Health-related quality of life

The EORTC-QLQ-C30 (version 3.0) was used to assess HRQoL [19]. In this study, only the five functioning scales (i.e., physical, role, cognitive, emotional, and social functioning) and the global health status/quality of life scale were used. Items are scored on a four-point Likert scale, which ranges from (1) *not at all* to (4) *very much*, except for the global quality of life scale, which ranges from (1) *very poor* to (7) *excellent*. Scores were linearly transformed to a 0–100 scale [20], with higher scores indicating a better functioning and better quality of life.

### Statistical analyses

NCR data on sociodemographic and clinical characteristics enabled us to compare respondents, non-respondents, and those with unverifiable addresses, using *t*-tests for continuous variables and chi-square (or Fisher’s exact) tests for categorical variables. In further analyses, only chemotherapy-treated survivors were included, as we are specifically interested in chemotherapy-induced peripheral neuropathy.

Using either *t*-tests or analyses of variance for continuous variables and chi-square tests for categorical variables, sociodemographic and clinical characteristics were compared between (1) survivors with painful CIPN either with or without non-painful CIPN, (2) survivors with non-painful CIPN only, and (3) survivors without any sensory CIPN.

Spearman’s correlation analysis was used to test the correlation between painful CIPN and non-painful CIPN within hands and feet, for numbness and tingling separately. Then, multivariate logistic regression analyses were conducted to identify sociodemographic, clinical, and psychological characteristics that were associated with painful CIPN and non-painful CIPN. The small number of survivors with painful CIPN did not permit us to include all a priori defined factors in the analyses. Therefore, factors were included based upon significant differences between survivors with painful CIPN, either with or without non-painful CIPN, survivors with non-painful CIPN only, and survivors without any sensory CIPN. Analyses in which age, sex, diabetes mellitus, and rheumatoid arthritis were also included yielded no other significant findings.

Finally, using analysis of covariance, EORTC QLQ-C30 mean scores were compared between (1) survivors with painful CIPN, either with or without non-painful CIPN, (2) survivors with non-painful CIPN only, and (3) survivors without any sensory CIPN. Confounding background variables included for adjustment were determined a priori and chosen to be age at time of questionnaire, sex, years since diagnosis, partner status, stage, number of comorbid conditions, diabetes mellitus, rheumatoid arthritis, osteoarthritis, and cancer type. Sensitivity analyses were then conducted to first compare survivors with numbness to those with tingling to examine whether numbness and tingling can indeed be evaluated together when looking at the impact on HRQoL. Secondly, survivors with painful CIPN only were compared to survivors with non-painful CIPN only. This was done to examine whether any found difference between painful CIPN, with or without non-painful CIPN, versus non-painful CIPN only, can be explained by the difference in symptom experience, or by the burden of experiencing multiple symptoms. Given the small sample size, this analysis was only corrected for age at time of diagnosis, years since diagnosis, number of comorbid conditions, and cancer stage. Clinically important differences were determined using the EORTC QLQ-C30 guidelines as proposed by Cocks et al. [21]. For example, for the “global quality of life” scale, a mean difference of 4–10 points, 10–15 points, and > 15 points is, respectively, considered a small, medium, and large clinically important difference.

All tests were two-sided and considered to be significant if *p* < 0.05. All statistical analyses were performed using SPSS 22 (IBM SPSS Statistics for Windows, Version 22.0 Armonk, NY: IBM Corps USA).

## Results

### Sample characteristics

For this study, data collected during the second data wave of a study among CRC survivors was used, which had a response rate of 83% (*n* = 1643). No differences in sociodemographic and clinical characteristics between respondents, non-respondents, and those with non-verifiable addresses were found (data not shown). Among survivors who were treated with chemotherapy (*n* = 500, 30%), data on painful CIPN and non-painful CIPN were available for 477 survivors.

CRC survivors with painful CIPN (*n* = 44, 9%) were diagnosed more recently and reported more anxiety and depressive symptoms compared to survivors without any sensory CIPN (*n* = 362, 76%) (Table 1). No differences in characteristics were found between survivors with painful CIPN and survivors with non-painful CIPN only (*n* = 70, 15%). Finally, compared to survivors who reported having neither painful nor non-painful CIPN, those with non-painful CIPN only were diagnosed more recently, more often diagnosed with colon cancer instead of rectal cancer, more often treated with radiotherapy, and they had a lower disease stage at diagnosis. Furthermore, they more often reported having osteoarthritis and they reported more anxiety symptoms.

**Table 1:** Sociodemographic and clinical characteristics of the chemotherapy-treated colorectal cancer survivors stratified by chemotherapy-induced peripheral neuropathy symptoms

	CRC survivors with painful CIPN^a^ (*n* = 45, 9%)	CRC survivors with non-painful CIPN only (*n* = 70, 15%)	CRC survivors without any sensory CIPN symptoms (362, 76%)	*p* value^b^
Age at time of survey (mean (SD))	65.9 (8.9)	66.1 (8.6)	66.6 (10.1)	0.91
Sex (female)	21 (47%)	26 (37%)	152 (42%)	0.31
Partner (yes)	39 (87%)	61 (87%)	292 (81%)	0.94
Educational level^c^				0.54
Low	8 (18%)	8 (11%)	48 (13%)	
Middle	26 (59%)	42 (60%)	222 (62%)	
High	10 (23%)	20 (29%)	91 (25%)	
Employed (yes)	7 (16%)	19 (27%)	85 (24%)	0.16
Number of comorbid conditions				0.85
None	7 (16%)	13 (20%)	108 (32%)	
One	15 (35%)	24 (36%)	112 (33%)	
Two or more	21 (49%)	29 (44%)	117 (35%)	
Tumor location				0.64
Colon	33 (73%)	54 (77%)*	228 (63%)	
Rectal	12 (27%)	16 (23%)	134 (37%)	
Years since diagnosis				0.47
Mean (SD)	4.4 (2.0)***	4.1 (1.7)***	6.0 (2.8)	
Range	2–11	2–9	2–12	
TNM stage				0.22
I	2 (4%)	0 (0%)*	24 (7%)	
II	4 (9%)	5 (7%)	59 (16%)	
III	33 (73%)	58 (83%)	242 (67%)	
IV	6 (13%)	5 (7%)	26 (7%)	
Unknown	0 (0%)	2 (3%)	11 (3%)	
Tumor differentiation grade				0.80
Well differentiated	5 (11%)	6 (9%)	34 (9%)	
Moderately differentiated	29 (64%)	41 (59%)	220 (61%)	
Poorly differentiated	6 (13%)	12 (17%)	47 (13%)	
Unknown	5 (11%)	11 (15%)	61 (17%)	
Radiotherapy (yes)	9 (20%)	11 (16%)**	116 (32%)	0.55
Comorbidities associated with PN-like symptoms^d^				
Diabetes mellitus	6 (14%)	8 (12%)	51 (15%)	0.78
Rheumatoid arthritis	3 (7%)	3 (5%)	21 (6%)	0.68
Osteoarthritis	10 (23%)	23 (35%)*	70 (21%)	0.20
Anxiety (mean (SD))	5.8 (4.2)*	5.7 (4.0)**	4.3 (3.5)	0.88
Depression (mean (SD))	5.6 (4.2)*	4.7 (3.4)	4.2 (3.6)	0.21

Some variables exceed 100% due to rounding off

CIPN, chemotherapy-induced peripheral neuropathy; SD, standard deviation

^a^CIPN survivors with painful CIPN were either with or without non-painful CIPN

^b^*P* values represent differences between CRC survivors with painful CIPN, either with or without non-painful CIPN, versus CRC survivors with non-painful CIPN only

^c^Education: low (no or primary school); medium (lower general secondary education or vocational training); high (pre-university education, high vocational training, university)

^d^Most frequent comorbidities associated with peripheral neuropathy

*p < 0.05 **p < 0.01 ***p < 0.001; *p* value represents difference between either CRC survivors with painful CIPN or CRC survivors with non-painful CIPN only versus CRC survivors without these symptoms

### Relationship between painful CIPN and non-painful CIPN

Of the 477 survivors with data on painful CIPN and non-painful CIPN, 103 survivors (22%) reported non-painful CIPN, irrespective of having painful CIPN or not (Table 2). Painful CIPN was reported by 45 survivors (9%), of which 25% (*n* = 11) reported painful CIPN only and 75% (*n* = 33) reported both painful and non-painful CIPN. In addition, one survivor reported painful CIPN but had missing data on non-painful CIPN.

**Table 2:** Presence of painful and non-painful CIPN in chemotherapy-treated colorectal cancer survivors

		Pain		Total
		No	Yes	
Non-painful CIPN	No	362 (76%)	11 (2%)	373
	Yes	70 (15%)	33 (7%)	103
Total		432	44*	

CIPN, chemotherapy-induced peripheral neuropathy

*One CRC survivor reported painful CIPN but had missing data on non-painful CIPN

Moderate correlations were observed between numbness and tingling within hands (*r*_s_ = 0.53) and within feet (*r*_s_ = 0.63). The correlations between shooting/burning pain and either numbness (*r*_s_ = 0.37 and *r*_s_ = 0.40) or tingling (*r*_s_ = 0.37 and *r*_s_ = 0.45) were weaker, both within hands and feet, respectively.

### Correlates of painful CIPN and non-painful CIPN

Identifying sociodemographic, clinical, and psychological factors independently associated with painful CIPN revealed that only a longer time since diagnosis was related to a decreased risk of reporting painful CIPN (Table 3). Painful CIPN was reported by 13% (*n* = 29) of survivors diagnosed 4–6 years ago, 6% (*n* = 4) of those diagnosed 6–8 years ago, 4% (*n* = 3) of those diagnosed 8–10 years ago, and 2% (*n* = 1) of those diagnosed 10–12 years ago.

**Table 3:** Correlates of painful and non-painful chemotherapy-induced peripheral neuropathy (CIPN) vs. no sensory CIPN in colorectal cancer survivors

	Painful CIPN		Non-painful CIPN	
	OR (95% CI)	*P* value	OR (95% CI)	*P* value
Years since diagnosis	0.78 (0.67–0.91)	0.001	0.67 (0.57–0.78)	< 0.001
Cancer stage (III and IV vs. I and II)	1.78 (0.67–4.86)	0.26	3.60 (1.25–10.34)	0.02
Tumor type (rectal)	1.46 (0.45–4.71)	0.53	1.58 (0.57–4.38)	0.38
Osteoarthritis (yes)	1.16 (0.52–2.56)	0.72	2.44 (1.29–4.63)	0.006
Radiotherapy (yes)	0.54 (0.14–2.03)	0.36	0.39 (0.12–1.28)	0.12
Anxiety	1.06 (0.95–1.19)	0.29	1.13 (1.02–1.26)	0.04
Depression	1.05 (0.94–1.17)	0.39	0.94 (0.84–1.05)	0.25

CIPN, chemotherapy-induced peripheral neuropathy; OR, odds ratio; 95% CI, 95% confidence interval

For non-painful CIPN, a higher disease stage (i.e., stage III/IV vs. I and II), osteoarthritis, and more anxiety symptoms were associated with an increased risk, while a longer time since diagnosis was associated with a decreased risk of having non-painful CIPN. Non-painful CIPN was reported by 31% (*n* = 67) of survivors diagnosed 4–6 years ago, 26% (*n* = 21) of those diagnosed 6–8 years ago, 13% (*n* = 8) of those diagnosed 8–10 years ago, and 8% (n = 6) of those diagnosed 10–12 years ago.

### Associations with health-related quality of life

**Fig. 1:**
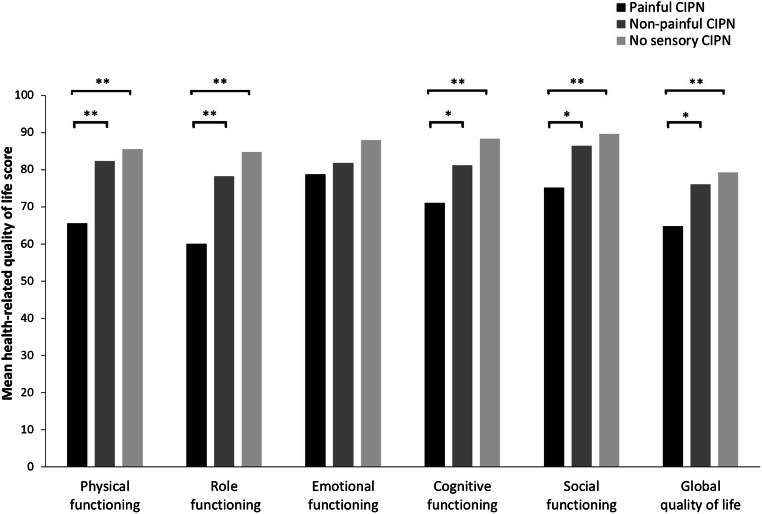
Comparison of health-related quality of life between colorectal cancer survivors with painful CIPN, survivors with non-painful CIPN only, and survivors with no sensory CIPN. *p < 0.01; **p < 0.001. Analyses were adjusted for age, sex, years since diagnosis, partner, cancer stage, number of comorbid conditions, diabetes mellitus, rheumatoid arthritis, osteoarthritis, and cancer type (rectal vs. colon cancer)

Among chemotherapy-treated CRC survivors, those with painful CIPN reported worse global quality of life and worse physical, role, cognitive, and social functioning compared to both survivors with non-painful CIPN and survivors without any sensory CIPN. All differences were of medium clinical relevance, except for the difference in role functioning between survivors with painful CIPN and those with non-painful CIPN, which was of small clinical relevance, and the difference in cognitive functioning between survivors with painful CIPN and those without any sensory CIPN, which was of large clinical relevance. No differences were found in emotional functioning. Also, no significant differences were observed between survivors with non-painful CIPN only and those without any sensory CIPN (Fig. 1).

Sensitivity analyses were conducted to first compare survivors with numbness (*n* = 8) and those with tingling (*n* = 36) (e.g., mean physical functioning = 91 vs. 82, respectively). No differences between these two groups were found in global quality of life or any of the functional scales (not formally tested because of small numbers).

Second, survivors with painful CIPN only (*n* = 11) were compared to survivors with non-painful CIPN only (*n* = 70). Survivors who experienced painful CIPN reported worse physical (55 vs. 82, *p* = 0.002), role (55 vs. 78, *p* = 0.01), and social (67 vs. 87, *p* = 0.003) functioning compared to those with non-painful CIPN only. The difference in role functioning was of medium clinical relevance, while the difference in physical and social functioning were of large clinical relevance. Finally, no differences in global quality of life (61 vs. 76, *p* = 0.05), emotional (77 vs. 82, *p* = 0.72), or cognitive functioning (65 vs. 81, *p* = 0.09) were observed.

## Discussion

In this secondary analysis of a study among long-term CRC survivors, we showed that the majority of survivors who reported painful CIPN, also reported non-painful CIPN, while having only painful CIPN was much less common. The prevalence of both painful and non-painful CIPN found in this study is comparable to the prevalence (9% and 26%, respectively) found in a previous study among CRC patients [13]. As the CRC survivors in this study were on average 5.6 years after diagnosis, this shows that both painful and non-painful CIPN are chronic problems that occur in a large number of CRC patients after chemotherapy.

Regarding the correlates of painful CIPN, only a longer time since diagnosis was significantly associated with a decreased risk, indicating that painful CIPN fades or even disappears over time in most patients. For non-painful CIPN, several correlates were found. First, a longer time since diagnosis was also associated with a decreased risk of non-painful CIPN. In addition, a higher disease stage, osteoarthritis, and more anxiety symptoms were associated with an increased risk of non-painful CIPN. The finding that osteoarthritis was a significant correlate is most likely because pain, numbness, and tingling are also frequently reported symptoms in that condition. Regarding anxiety, several other studies also found an association between anxiety and CIPN [12, 22–25]. As the current study is cross-sectional, we cannot draw conclusions on the direction of this association. It could be that CIPN symptoms result in more anxiety, due to the pain and limitations in daily functioning [26]. However, two previous studies found that pre-treatment anxiety was associated with neuropathic symptoms, but not to pain in hands or feet [12], and persistent CIPN [25]. A psychological explanation could be that people who are anxious often develop a catastrophic thinking style and that catastrophizing and anxiety sensitivity could be a common vulnerability factor for chronic CIPN [27]. Pro-inflammatory cytokines may offer a biological explanation, as they have been linked to both anxiety and CIPN [28, 29]. Also, recovery from the nerve injury in CIPN could be slowed down by the pro-inflammatory cytokines that are produced due to the anxiety. In addition, inflammatory mediators (produced due to anxiety) can contribute to hypersensitivity through stimulation or potentiation of nociceptive transduction at peripheral terminals and central changes [30].

In this study, the association between (non-)painful CIPN and HRQoL was also examined. We found that painful CIPN was related to a worse HRQoL compared to those with non-painful CIPN only and those with no sensory CIPN. This finding is in line with prior studies done in diabetes research [11]. In the previously mentioned study among CRC patients, only non-painful CIPN was associated with higher odds of having poor HRQoL at baseline, while only painful CIPN was associated with declining HRQoL assessed 4–5 weeks later [13]. In our study, no differences in HRQoL were found between survivors with non-painful CIPN only and those without sensory CIPN. It should be noted that, while survivors in the “no sensory CIPN” group did not report painful or non-painful (i.e., numbness and tingling) CIPN, they could still experience neuropathy symptoms, as we did not exclude those who reported other neuropathy symptoms measured by the CIPN20. However, as one of our previous studies using the same sample [5] showed no differences between survivors with and without chemotherapy on any of these symptoms, the findings regarding HRQoL among those with non-painful CIPN most likely indicate that they have a similar HRQoL to those who have neuropathy symptoms related to normal aging [31] and cancer itself [32].

While painful CIPN has a large impact on HRQoL, there are few effective treatments for it. Duloxetine is currently the only drug that has shown to be effective in the treatment of painful CIPN [33]. Anticonvulsants and other antidepressants, which have shown their efficacy in other neuropathic pain populations, are also used to treat CIPN. However, given the side effects and lack of efficacy, adherence is often poor [34]. Cognitive-behavioral therapy (CBT) might also improve HRQoL in patients with neuropathic pain [35]. A recent pilot study tested the effectiveness of a self-guided online CBT for painful CIPN, with promising results [36]. However, as the development of (severe) CIPN during chemotherapy often leads to a reduction in chemotherapy doses, or even cessation of treatment [3], it also seems crucial for future studies to focus more on the prevention of CIPN.

Several limitations of our study need to be acknowledged. First, we do not have information on the presence of neuropathy symptoms (e.g., idiopathic or entrapment neuropathy) before chemotherapy. While we did control for diabetes, rheumatoid arthritis, and osteoarthritis, which could lead to neuropathy-like symptoms, we cannot be certain that the neuropathy symptoms assessed in this study were caused by the chemotherapy. We also did not have any information on the type of chemotherapeutic agent, number of chemotherapy cycles, and possible dose reduction, while it is known that CIPN depends on these factors and thereby could have impacted HRQoL [3]. Therefore, to be able to make recommendations on treatment decisions and alterations to reduce painful CIPN, future studies should include these measures. It should be noted that oxaliplatin, a chemotherapeutic agent often associated with CIPN [37], has been used since 2007, which is the last 2 years of our study period. This could be a confounder when looking at the prevalence of (non-)painful CIPN in our study. Another limitation is that we did not perform a clinician-based assessment of CIPN, while it was concluded in a recent review on the clinical assessment tools for CIPN that patient-reported assessments of CIPN should be combined with clinician-rated neurological assessment tools to be able to give a clear picture of neuropathy [38]. Also, given the rather low sample size of survivors with painful CIPN, we could not include many possible correlates. It could be that important factors not included in the analysis are associated with painful CIPN. Furthermore, some factors that were included in the analysis, particularly those significant in non-painful CIPN, might have been significant within a larger sample. Consequently, the question remains whether there are correlates that apply to both painful and non-painful CIPN or whether there are correlates that are unique to painful CIPN (or non-painful CIPN), making it possible to distinguish between the two. Therefore, in future research, a larger sample should be included so a more thorough examination of the differences between subgroups (i.e., patients with painful CIPN only, patients with both painful and non-painful CIPN, and patients with non-painful CIPN only) can be done. Finally, it should be acknowledged that we did not correct for multiple testing, thereby increasing the potential for false positive findings.

Despite these limitations, this is, to the best of our knowledge, the first study that has examined the prevalence, correlates, and impact of painful versus non-painful CIPN on HRQoL among long-term CRC survivors. The findings of this study indicate that painful CIPN must be distinguished from other CIPN symptoms, such as non-painful CIPN, given the large impact on HRQoL. Therefore, patients should be informed about the relationship between painful CIPN and HRQoL. Moreover, patients should also be screened specifically on painful CIPN, as treatment options, while limited, are available for painful CIPN. Finally, the question remains whether painful CIPN should be distinguished from non-painful CIPN when looking at predictors, mechanisms and therefore also possible treatment.
